# ACAT1 promotes proliferation and metastasis of bladder cancer via AKT/GSK3β/c-Myc signaling pathway

**DOI:** 10.7150/jca.95549

**Published:** 2024-04-23

**Authors:** Tingjun Wang, Gang Wang, Danni Shan, Yayun Fang, Fenfang Zhou, Mengxue Yu, Lingao Ju, Gang Li, Wan Xiang, Kaiyu Qian, Yi Zhang, Yu Xiao, Xinghuan Wang

**Affiliations:** 1 Department of Urology, Laboratory of Precision Medicine, Zhongnan Hospital of Wuhan University, Wuhan, China.; 2 Department of Biological Repositories, Human Genetic Resources Preservation Center of Hubei Province, Hubei Key Laboratory of Urological Diseases, Zhongnan Hospital of Wuhan University, Wuhan, China.; 3 Department of Radiology, Zhongnan Hospital of Wuhan University, Wuhan, China.; 4 Euler Technology, ZGC Life Sciences Park, Beijing, China.; 5 Center for Quantitative Biology, School of Life Sciences, Peking University, Beijing, China.; 6 Medical Research Institute, Frontier Science Center for Immunology and Metabolism, Taikang Center for Life and Medical Sciences, Wuhan University, Wuhan, China.

**Keywords:** bladder cancer, ACAT1, AKT/GSK3β/c-Myc, proliferation, metastasis

## Abstract

Acetyl-CoA acetyltransferase 1 (ACAT1) plays a significant role in the regulation of gene expression and tumorigenesis. However, the biological role of ACAT1 in bladder cancer (BLCA) has yet to be elucidated. This research aimed to elucidate the bioinformatics features and biological functions of ACAT1 in BLCA. Here, we demonstrate that *ACAT1* is elevated in BLCA tissues and is correlated with specific clinicopathological features and an unfavorable prognosis for survival in BLCA patients. *ACAT1* was identified as an independent risk factor in BLCA. Phenotypically, both *in vitro* and* in vivo*, *ACAT1* knockdown suppressed BLCA cell proliferation and migration, while ACAT1 overexpression had the opposite effect. Mechanistic assays revealed that ACAT1 enhances BLCA cell proliferation and metastasis through the AKT/GSK3β/c-Myc signaling pathway by modulating the cell cycle and EMT. Taken together, the results of our study reveal that ACAT1 is an oncogenic driver in BLCA that enhances tumor proliferation and metastasis, indicating its potential as a diagnostic and therapeutic target for this disease.

## Introduction

Bladder cancer (BLCA), which originates from the bladder mucosa, is one of the most prevalent malignant tumors affecting the urinary system 1. It poses a considerable global public health threat, accounting for 213,000 deaths and 573,278 new cases annually 1-3. The treatment paradigm for BLCA has evolved from the conventional combination of surgery and chemotherapy to a comprehensive approach involving surgery, radiotherapy, chemotherapy, and immunotherapy 4-6.

Metastatic BLCA is usually difficult to cure completely, and systemic therapy, including chemotherapy, immunotherapy, and targeted therapy, is the main treatment for such patients. For patients with metastatic disease who can tolerate cisplatin chemotherapy, cisplatin-based combination chemotherapy is the first-line standard treatment 6. The median OS was 14 months and 15.2 months for patients receiving MVAC treatment with methotrexate, vinblastine, adriamycin and cisplatin and for patients receiving GC treatment with gemcitabine and cisplatin, respectively 7. The lower toxicity of GC compared to standard MVAC makes GC the standard regimen 8. Carboplatin plus gemcitabine is considered the standard first-line regimen for metastatic patients who cannot tolerate cisplatin chemotherapy 6. An overall response rate (ORR) of 42% was reported for patients receiving carboplatin plus gemcitabine 9, 10. The Food and Drug Administration (FDA) (but not the European Medicines Agency (EMA)) has approved pembrolizumab and atezolizumab as first-line regimens for patients who are not suitable for any platinum-based chemotherapy 11, 12. The study revealed that metastatic patients who received pembrolizumab had an ORR of 28.9% and a 6-month OS of 67%, compared with an ORR of 24% and a median OS of 16.3 months for those who received atezolizumab 13. The progression free survival (PFS) of patients with metastatic BLCA was 7 months, and the median OS was 15 months 14. The most common metastatic sites were bone, lung, liver and brain, and the average survival times of these metastatic patients were 9 months, 10.2 months, 7.1 months, and 7.3 months, respectively 15.

Despite these advances, BLCA exhibits a high recurrence rate and a propensity for progression, necessitating regular follow-up and review and imposing a substantial economic burden on patients 16, 17. Consequently, investigating the biological mechanisms underlying BLCA occurrence and development, identifying novel biomarkers, and uncovering potential therapeutic targets are crucial for the diagnosis, treatment, and prognosis of BLCA.

Post-translational modifications, particularly acetylation, have been documented in BLCA 18. Notably, attention has been given to ACAT1, an enzyme localized in the mitochondrion that was initially recognized for its pivotal role in the rare genetic disease β-ketothiolase deficiency. Subsequent research revealed that ACAT1 facilitates a metabolic switch in cancer cells, alternating between glycolysis and oxidative phosphorylation by acetylating PDP1 and PDHA1. This adaptive response to external changes ultimately promotes tumor proliferation 19, 20. Further studies underscore the close relationship between ACAT1 and malignant tumors. In colorectal cancer, ACAT1 acetylates ME1 or regulates the utilization of β-hydroxybutyrate, thereby promoting cancer growth 21, 22. In prostate cancer, ACAT1 is upregulated in high-grade cases. ACAT1 knockdown renders castration-resistant prostate cancer cells more susceptible to enzalutamide treatment 23, 24. It was discovered that MAPK pathway activity was enhanced by SIRT5 via ACAT1, thereby boosting the migration, invasion, and proliferation ability of prostate cancer cells 25. Additionally, reports indicate increased ACAT1 levels in prostate cancer tissues of patients exposed to organic substances such as dioxins and polychlorinated biphenyls, potentially promoting cancer cell growth and metastasis 26. ACAT1 was found to be upregulated in doxorubicin-resistant uterine cancer, and its knockdown correlated with inhibited migration and proliferation of cancer cells, as well as enhanced apoptosis 27. In liver cancer, ACAT1 acetylates GNPAT, stabilizing FASN and promoting lipid metabolism, thereby contributing to hepatocarcinogenesis 28.

Despite these previous findings, the biological role of ACAT1 in BLCA has largely been elusive. In this study, we confirmed not only that ACAT1 promoted the proliferation and migration of BLCA cells both *in vivo* and *in vitro* but also that ACAT1 enhanced BLCA cell proliferation and metastasis via the AKT/GSK3β/c-Myc signaling pathway by modulating the cell cycle and EMT. These findings provide novel insights for identifying new biomarkers and potential therapeutic targets for BLCA.

## Materials and methods

### Human bladder cancer tissue

The study using human bladder cancer tissues and paired paracancerous tissues (n = 9) was approved by the Institutional Ethics Committee of Zhongnan Hospital of Wuhan University (approval number: 2021125). Informed consent was obtained from all individuals. Bladder cancer and paired paracancerous tissues were collected after radical bladder cancer surgery and pathological confirmation. The tissue chips for the present study were kindly supplied by Shanghai OUTDO Biotech Co., Ltd., Shanghai, China.

### Cell culture experiments

BLCA cell lines (UM-UC-3, T24, and 5637) were obtained from the Chinese Academy of Sciences, Shanghai, China. T24 and 5637 cells were cultured and passaged in RPMI-1640 medium (Gibco, USA), and UM-UC-3 cells were maintained in MEM (Gibco, USA). All cell culture media were supplemented with 10% fetal bovine serum (FBS) (Excell Bio, China). All cell lines were authenticated by STR profiling. Cell culture experiments were performed in an incubator at 37°C in humidified conditions containing 95% air and 5% CO_2_.

### siRNAs and plasmid transfection

ACAT1-specific siRNA was obtained from GenePharma (Suzhou, China), and the FLAG-ACAT1 plasmid was obtained from the MiaoLing Plasmid Platform (Wuhan, China). The sequence information for the siRNAs is provided in Supplementary Table S4. Both siRNA and plasmid transfections were performed according to the Lipofectamine 3000 Reagent protocol (Invitrogen, L3000015).

### Total mRNA extraction and quantitative reverse transcription PCR (qRT-PCR)

Total mRNA was extracted using the HiPure Total RNA Mini kit (Magen, R4111-03). qRT-PCR was performed using iTaq Universal SYBR Green Supermix (Bio-Rad, #1725125), and the primer sequences are provided in Supplementary Table S5.

### RNA-seq analysis

Total RNA extracted from the samples was quantified, and a library for stranded RNA sequencing was prepared using 2 μg of RNA. Sequencing was performed using an Illumina NovaSeq 6000 platform. Trimmomatic (version 0.36) was used to filter the raw data, and STAR software (version 2.5.3) was used to map the clean reads to the human genome (assembly GRCh38). The featureCounts were used to count the reads mapped to gene exon regions before the calculation of the RPKM.

### Western blot

Cells were lysed in RIPA (Beyotime, P0013B) buffer supplemented with phosphatase and protease inhibitors. The protein lysates were centrifuged, separated via SDS-PAGE, transferred to PVDF membranes, and blocked with 5% skim milk in TBST buffer. Primary antibodies were incubated overnight at 4°C, followed by secondary antibody incubation, and protein signals were measured using a chemiluminescence kit (Bio-Rad, USA). Primary antibody information is provided in Supplementary Table S6.

### MTT assays

A total of 3,000 transfected cells per well were seeded in 96-well plates. Then, 20 μL of MTT solution was added to each well every other day. After incubating for 4 hrs, the supernatant was discarded, and 200 μL of DMSO solution was added to each well to fully dissolve the precipitate. After mixing with shaking on a shaker for 10 mins, a microplate reader (SpectraMax M2, USA) was used to measure the absorbance. The assay was carried out as described above for five consecutive days.

### Colony formation assays

Transfected cells (1000 per well) were added to 6-well plates and cultured for approximately ten days until macroscopic colonies formed. After the culture medium was discarded, the cells were fixed for 1 hr in 4% formaldehyde, stained for 1 hr in 0.1% crystal violet, and subsequently washed and dried.

### Migration assays

The Transwell chambers were filled with a set number of transfected cells (UM-UC-3 or T24 cells: 4 × 10^4^ cells per well; 5637 cells: 1.2 × 10^5^ cells per well). Serum-free medium was added to the upper chamber, and medium containing 10% FBS was added to the lower chamber. The cells that remained on the side of the upper chamber were gently rubbed with cotton swabs after incubating for 24 hrs. Before 0.1% crystal violet staining of the cells that crossed the membrane was performed, the cells were fixed in 4% paraformaldehyde.

### Wound healing assays

When the cells in the 6-well plates were 100% confluent, a scratch was made. Subsequently, the cells were washed with PBS, and the medium was replaced with serum-free medium for continued culture in a 37°C incubator. The scratched area was photographed at 0 hr and 24 hr. Gap closure rate (%) = (0 hr distance - 24 hr distance)/0 hr distance × 100%.

### Flow cytometry

The transfected cells were collected, washed three times with sterile PBS and subsequently centrifuged, after which the supernatant was discarded. Next, according to the protocol of the Cell Cycle Staining Kit (MultiSciences Biotech, CCS012), the cell samples were processed properly. Finally, a flow cytometer (Beckman Cytoflex) was used to analyze the samples.

### Establishment of stable *ACAT1*-knockdown cell lines

The lentiviruses LV-shNC and LV-shACAT1 were obtained from GenePharma (Suzhou, China). T24 cells were transfected with lentivirus, and positively infected cells were screened with 1 μg/mL puromycin. Immunoblotting and qRT-PCR confirmed the successful generation of stable *ACAT1*-knockdown cell lines.

### *In vivo* experiments

Male BALB/c nude mice (4 weeks old) were obtained from WQJX Biotechnology (Wuhan, China). The animals were housed under a 12-h light/dark cycle in a specific pathogen-free (SPF) environment. After seven days of adaptive feeding, the nude mice were randomly divided into groups for subsequent experiments. For subcutaneous tumor-bearing experiments, 4 × 10^6^ LV-shNC/LV-shACAT1 cells were resuspended in 100 μL of PBS and then subcutaneously transplanted. The size of the tumors was measured every three days. The tumor volume was estimated using the formula V=1/2 × L × S^2^ (L: long diameter, S: short diameter). Finally, tumor tissues were obtained from nude mice, weighed and fixed in 4% formaldehyde and subsequently subjected to immunohistochemistry and H&E staining. For lung metastasis experiments, 9 × 10^5^ LV-shNC/LV-shACAT1 cells were resuspended in 100 μL of PBS before tail vein injection. After approximately 1 month of normal feeding, the nude mice were anesthetized and analyzed for lung metastases by measuring their fluorescence intensity. We dissected and fixed the lung tissues in 4% formaldehyde, followed by H&E staining. In the aforementioned *in vivo* model, mice were randomly assigned, and no blinding was performed. The Laboratory Animal Welfare and Ethics Committee of Zhongnan Hospital of Wuhan University (approval number: ZN2023048) approved the nude mouse experiments, which were performed according to the relevant regulations.

### Immunohistochemical (IHC) and hematoxylin & eosin (H&E) staining

Tissue slices were deparaffinized and rehydrated with xylene, ethanol at different concentrations, and water for both H&E staining and IHC. After 15 mins in 10% hematoxylin and 10 mins in 1% eosin, the slices were stained with H&E. The anti-Ki67 antibody was incubated overnight with paraffin sections of mouse xenograft tumors at 4°C for IHC, followed by incubation for 50 mins each with secondary antibodies and DAB. Hematoxylin was used to stain the nuclei. The slices were dehydrated with xylene and different concentrations of ethanol. We used an automated slide scanning microscope for image acquisition and analysis. The antibody information is provided in Supplementary Table S6.

### Bioinformatics analysis

The mean optical density of the immunohistochemical images on the tissue chips was measured by Image-Pro Plus Version 6.0. Supplementary Fig. S1A: RNA-seq data and matching clinical information for 33 kinds of tumors were obtained from The Cancer Genome Atlas (TCGA) database. *p* values, hazard ratios (HRs), and 95% confidence intervals (CIs) were calculated via univariate Cox survival analysis via the "forestplot" package of R software. Fig. 2A: The mRNA expression matrix of bladder cancer cells was obtained from the Cancer Cell Line Encyclopedia (CCLE) database. The data analysis was performed using the “ggplot2” package (version 3.3.3) of R software. Supplementary Fig. S2A, S2F; Fig. 5B, 6B: RNA-seq data and matching clinical information for bladder cancer patients were obtained from the TCGA database. The gene set variation analysis (GSVA) package of R software, which selects “ssgsea” as a parameter method, was used to gather and analyze the genes included in the corresponding pathway. Finally, Spearman correlation was performed to analyze the correlations among genes and pathway scores. R software version 4.0.3 was used for all the above bioinformatics analyses. *p* < 0.05 was considered to indicate statistical significance.

### Gene set enrichment analysis (GSEA)

RNA-seq data from *ACAT1* knockdown cells and public datasets obtained from ArrayExpress, TCGA, and GEO database were analyzed using GSEA (version 4.2.3). Absolute values of normalized enrichment score (NES) > 1 and *p <* 0.05 were considered to indicate statistical significance.

### Statistical analysis

In this study, all the assays were repeated at least three times. All the data are presented as the mean ± standard deviation (SD). The statistical significance of the differences between the two groups was evaluated by two-tailed Student's t test. One-way ANOVA was used to assess the statistical significance of the differences among the three groups. All the statistical analyses were performed with GraphPad Prism Version 8.0 and SPSS Version 23.0; ns: not significant; *: *p* < 0.05; **: *p* < 0.01; ***: *p* < 0.001.

## Results

### Elevation of ACAT1 in BLCA and its correlation with poor prognosis

**Figure 1:**
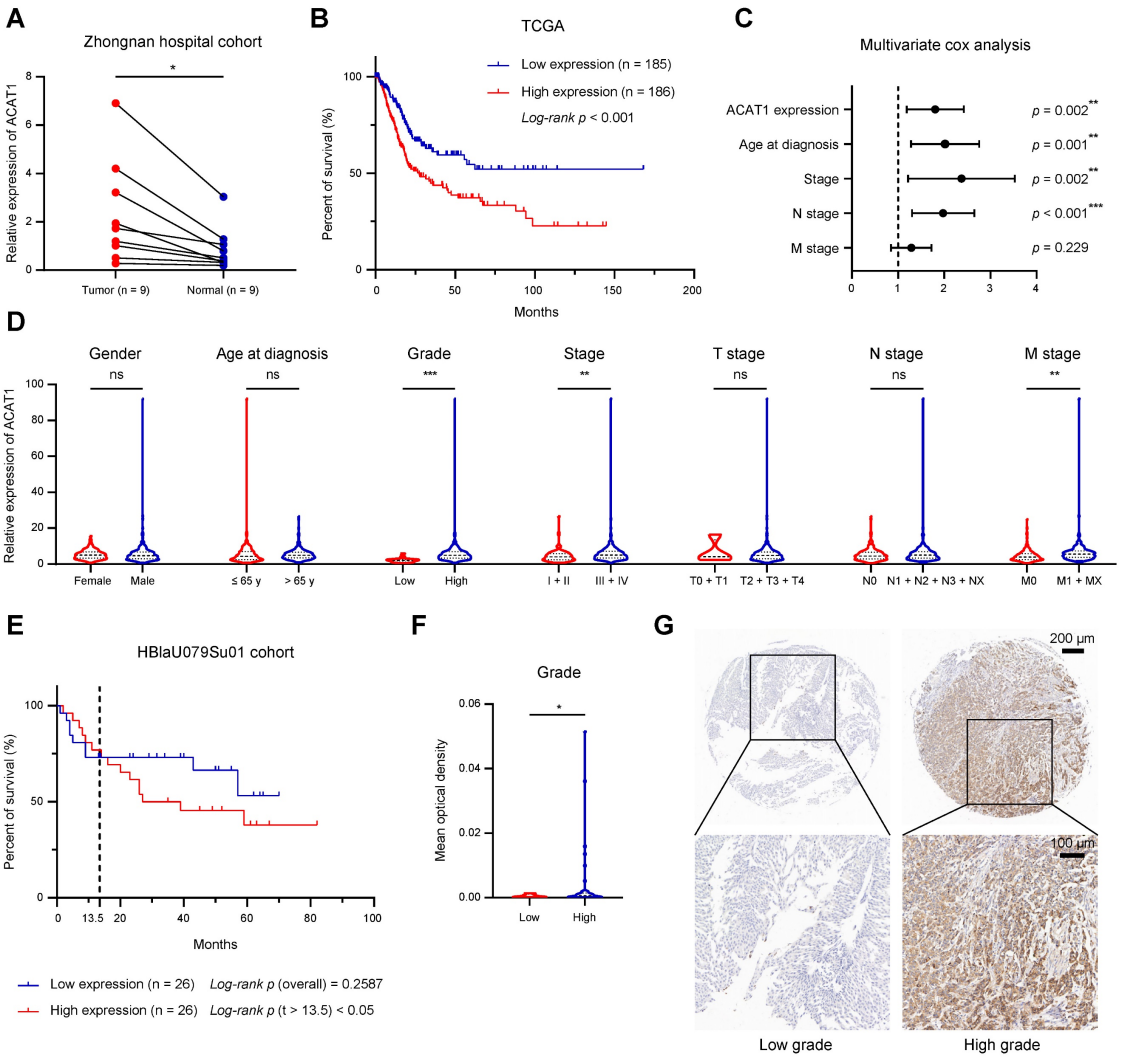
** ACAT1 was elevated in BLCA and indicated poor prognosis. (A)** Changes in *ACAT1* mRNA levels in BLCA tissues and paired adjacent tissues in the Zhongnan Hospital cohort (n = 9).** (B)** The prognostic curves (rates of overall survival) of patients with different *ACAT1* mRNA expression levels in the TCGA BLCA dataset were analyzed. **(C)** *ACAT1* expression, age at diagnosis, stage and N stage were found to be independent risk factors for poor survival in BLCA patients. **(D)** Differences in *ACAT1* mRNA expression levels in patients according to sex, age at diagnosis, grade, stage, T stage, N stage and M stage in the TCGA BLCA dataset. **(E)** The prognostic curves (overall survival rate) of patients with different ACAT1 protein expression levels in the HBlaU079Su01 cohort were analyzed. **(F**-**G)** In the HBlaU079Su01 cohort, ACAT1 protein expression was increased in patients with high-grade BLCA. The scale bars are 200 μm (top) and 100 μm (bottom); ns: not significant; *: *p* < 0.05; **: *p* < 0.01; ***: *p* < 0.001*.*

We examined the mRNA expression of *ACAT1* in 9 pairs of surgical specimens from BLCA tissues and matched paracancerous tissues. The analysis revealed significant upregulation of *ACAT1* mRNA in BLCA tissues (Fig. 1A). Univariate Cox survival analyses, in conjunction with Kaplan-Meier survival analysis of *ACAT1*, utilized data from 33 tumor types obtained from the TCGA database. These analyses revealed that *ACAT1* acted as an independent risk factor for BLCA, and its high mRNA expression was positively correlated with poor OS in BLCA patients (Fig. 1B and Supplementary Fig. S1A). Univariate and multivariate Cox survival analyses of BLCA patients revealed that independent risk factors, including *ACAT1* expression, age at diagnosis, stage, and N stage, were positively associated with poor survival in BLCA patients (Fig. 1C and Supplementary Table S1).

A chi-square test analyzing the association between *ACAT1* mRNA expression and the clinicopathological features of BLCA patients in the TCGA database indicated that *ACAT1* mRNA expression levels were positively related to tumor grade, stage, and M stage (Supplementary Table S2). Additionally, increased *ACAT1* mRNA expression was observed in patients with high-grade and high-stage BLCA, suggesting that the mRNA expression level of *ACAT1* was positively correlated with stage and grade (Fig. 1D). Furthermore, *ACAT1* mRNA expression was upregulated in tissues from patients with distant metastasis (Fig. 1D), suggesting the potential involvement of *ACAT1* in promoting epithelial-mesenchymal transition (EMT) in BLCA. Chi-square test examining the relationship between *ACAT1* mRNA expression and the clinicopathological features of BLCA patients in the GSE13507 dataset indicated that *ACAT1* mRNA expression was positively associated with the grade of BLCA (Supplementary Table S3). Moreover, increased *ACAT1* mRNA expression was observed in BLCA patients with high-grade disease, high-T stage disease, or muscle invasion, suggesting that *ACAT1* mRNA expression level was positively correlated with tumor grade and T stage and that *ACAT1* may promote muscle invasion (Supplementary Fig. S1B-D). In the HBlaU079Su01 cohort, poor overall survival was positively associated with high ACAT1 protein expression (Fig. 1E). Additionally, increased ACAT1 protein expression was observed in patients with high-grade BLCA (Fig. 1F-G). In conclusion, our findings demonstrated that ACAT1 is positively associated with poor prognosis and may function as an oncogene in BLCA.

### Knockdown of *ACAT1* slowed BLCA cell proliferation

**Figure 2:**
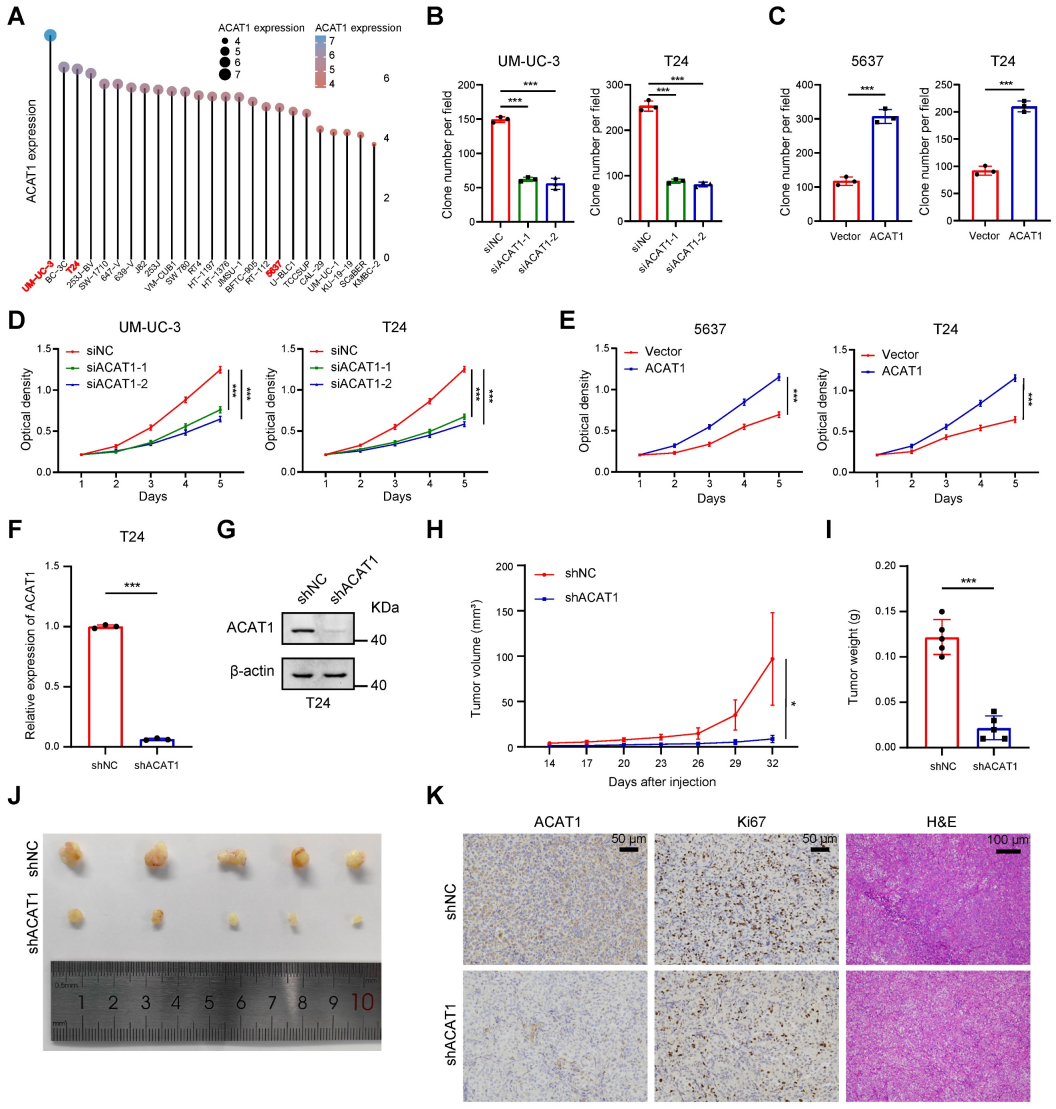
** Knockdown of *ACAT1* slowed the proliferation of BLCA cells. (A)** Distribution of mRNA expression in different BLCA cell lines. Dots of different colors and sizes represent different amounts of expression. **(B-C)** Statistical analysis of colony formation assays performed on BLCA cells after knockdown (UM-UC-3 and T24 cells) or overexpression (5637 and T24 cells) of ACAT1.** (D-E)** MTT assays were performed on BLCA cells after knockdown (UM-UC-3 and T24 cells) or overexpression (5637 and T24 cells) of ACAT1. **(F-G)** qRT-PCR and immunoblotting were used to evaluate the knockdown efficiency of shACAT1 in T24 cells. **(H)** Growth curves of xenograft tumors from the shNC and shACAT1 groups. **(I)** Measurement of xenograft tumor weight. **(J)** Anatomical gross images of xenograft tumors in the shNC and shACAT1 groups (n = 5). **(K)** Representative images of ACAT1 and Ki67 immunohistochemistry as well as H&E staining of xenograft tumors. The scale bars are 50 μm (left), 50 μm (middle), and 100 μm (right); ns: not significant; *: *p <* 0.05; **: *p* < 0.01; ***: *p* < 0.001.

For the selection of suitable BLCA cell lines for subsequent experiments, we referred to the CCLE database. Notably, *ACAT1* expression was relatively high in UM-UC-3 cells, low in 5637 cells, and intermediate in T24 cells among commonly used BLCA cell lines (Fig. 2A). Consequently, UM-UC-3 and T24 cells were chosen for the *ACAT1* knockdown experiments, while 5637 and T24 cells were selected for the overexpression reversion experiments.

GSVA was employed to process the data obtained from the TCGA database using R software, revealing a positive association between *ACAT1* and tumor proliferation (Supplementary Fig. S2A). Plasmids and siRNAs were utilized to upregulate and downregulate ACAT1 expression, respectively, in BLCA cells. Both the knockdown (in UM-UC-3 and T24 cells) and overexpression (in 5637 and T24 cells) efficiencies were assessed through qRT-PCR and Western blot assays (Supplementary Fig. S2B-C). Subsequently, we evaluated the impact of *ACAT1* knockdown on BLCA cell proliferation. Clonogenic and MTT assays demonstrated that *ACAT1* knockdown significantly reduced the proliferation of UM-UC-3 and T24 cells (Fig. 2B, 2D and Supplementary Fig. S2D). Conversely, ACAT1 overexpression promoted the growth of 5637 and T24 cells (Fig. 2C, 2E and Supplementary Fig. S2E).

To assess the *in vivo* impact of ACAT1 knockdown, lentivirus transfection was used to establish stable *ACAT1*-knockdown cell lines. The efficacy of *ACAT1* knockdown in T24 cells was validated using qRT-PCR and Western blot assays (Fig. 2F-G). Subcutaneously, T24 cell lines with *ACAT1* stable knockdown were subcutaneously implanted into nude mice to generate xenografts. After a designated period, the tumors that had formed under the skin of the nude mice were harvested. The tumors in the shACAT1 group exhibited a smaller volume and lighter weight than did those in the shNC group (Fig. 2H-J). IHC and H&E staining revealed decreased Ki67 expression in tumor tissues *from patients with* stable ACAT1 knockdown (Fig. 2K). Taken together, our findings showed that ACAT1 regulated the proliferation of BLCA cells.

### *ACAT1* knockdown attenuated BLCA cell migration

**Figure 3:**
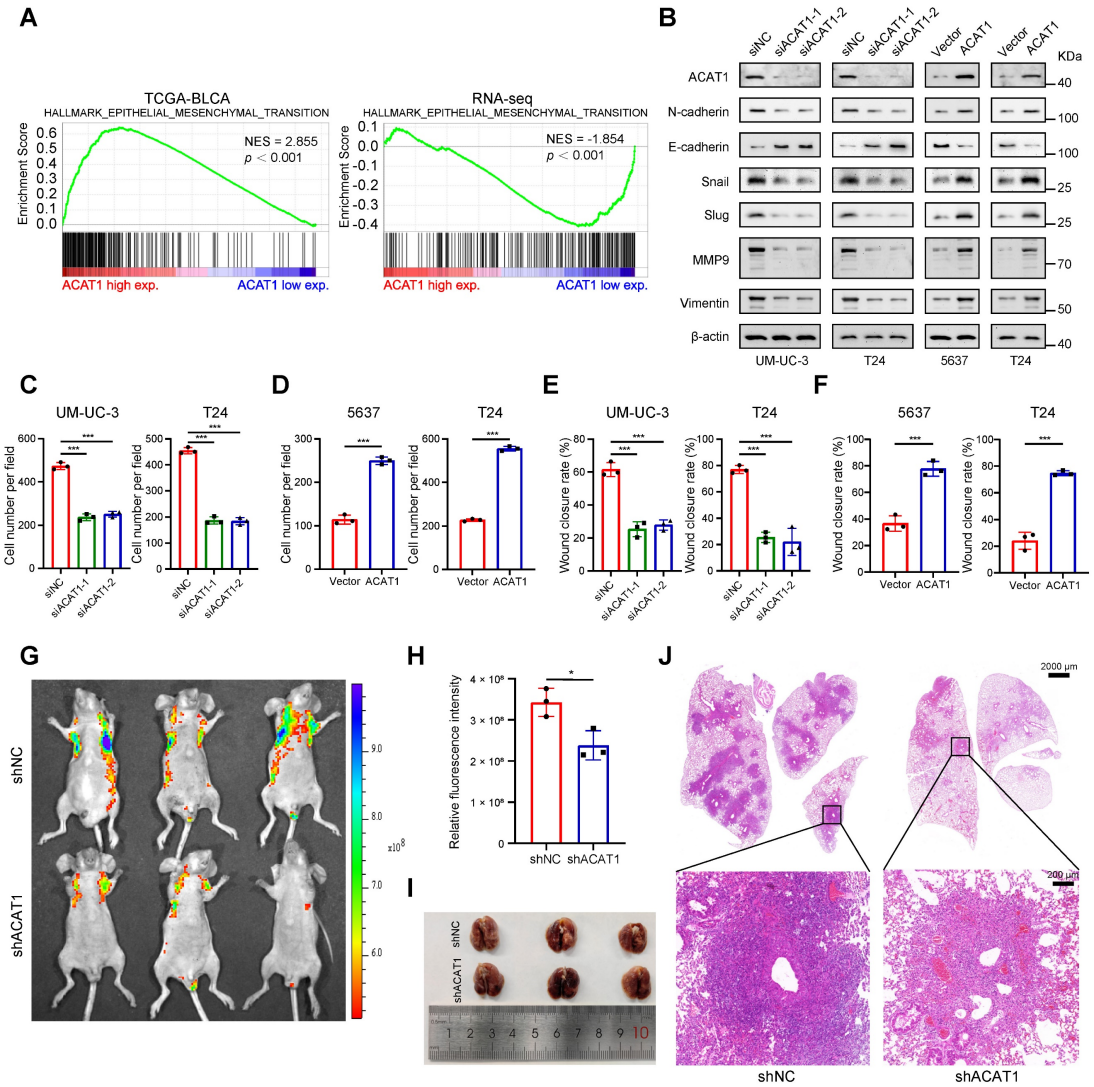
***ACAT1* knockdown attenuated BLCA cell migration. (A)** GSEA of the TCGA BLCA dataset and RNA-seq data showed enrichment in EMT. **(B)** Changes in the expression of EMT-related proteins after ACAT1 knockdown (UM-UC-3 and T24 cells) or overexpression (5637 and T24 cells) were measured via western blot assays. **(C-D)** Statistical analysis of transwell assays performed on BLCA cells after knockdown (UM-UC-3 and T24 cells) or overexpression (5637 and T24 cells) of ACAT1. **(E-F)** Statistical analysis of wound healing assays performed on BLCA cells after knockdown (UM-UC-3 and T24 cells) or overexpression (5637 and T24 cells) of ACAT1.** (G)** Lung fluorescence images were taken to detect lung metastasis formation in nude mice (n = 3) after injection of T24-LV-shNC/T24-LV-shACAT1 cells via the tail vein. **(H)** Measurement of lung metastasis fluorescence intensity. **(I-J)** Gross views of whole-lung anatomy and representative H&E-stained sections of lung tissues. The scale bars are 2000 μm (top) and 200 μm (bottom); ns: not significant; *: *p* < 0.05; **: *p* < 0.01; ***: *p* < 0.001.

GSVA indicated that *ACAT1* was positively associated with EMT (Supplementary Fig. S2F). Concurrently, GSEA revealed that EMT was enriched in the TCGA database and various Gene Expression Omnibus (GEO) datasets (GSE3167, GSE7476, GSE13507, GSE32894, and GSE48075), the UROMOL cohort and RNA-seq data (Fig. 3A and Supplementary Fig. S2G-H). Alongside the findings of previous reports from clinical samples, these bioinformatics findings suggest the potential involvement of ACAT1 in the EMT process in BLCA.

We next investigated whether ACAT1 regulates BLCA cell metastasis through EMT by immunoblotting. *ACAT1* knockdown decreased the expression of classical EMT markers, including N-cadherin, Snail, Slug, MMP9, and vimentin, while increasing the expression of E-cadherin. However, ACAT1 overexpression had the opposite effect (Fig. 3B). Transwell experiments were subsequently performed to assess the influence of *ACAT1* knockdown on the migration of BLCA cells. *ACAT1* knockdown impaired the migration of UM-UC-3 and T24 cells (Fig. 3C and Supplementary Fig. S3A), while ACAT1 overexpression enhanced the migration of 5637 and T24 cells (Fig. 3D and Supplementary Fig. S3B). Wound healing assays further validated the aforementioned results (Fig. 3E-F and Supplementary Fig. S3C-F).

To explore the impact of *ACAT1* knockdown on metastasis *in vivo*, BLCA cells stably transfected with LV-shNC or LV-shACAT1 were injected into nude mice through the tail vein. Lung metastasis models were established with this method. There was less lung metastasis in the shACAT1 group than in the shNC group (Fig. 3G-H), suggesting a reduction in lung metastasis in the shACAT1 group. Gross observation of lung tissues and H&E staining further confirmed these findings (Fig. 3I-J). Our results demonstrated that ACAT1 regulated the metastatic ability of BLCA cells.

### Knockdown of *ACAT1* caused G1 phase cell cycle arrest in BLCA cells

**Figure 4:**
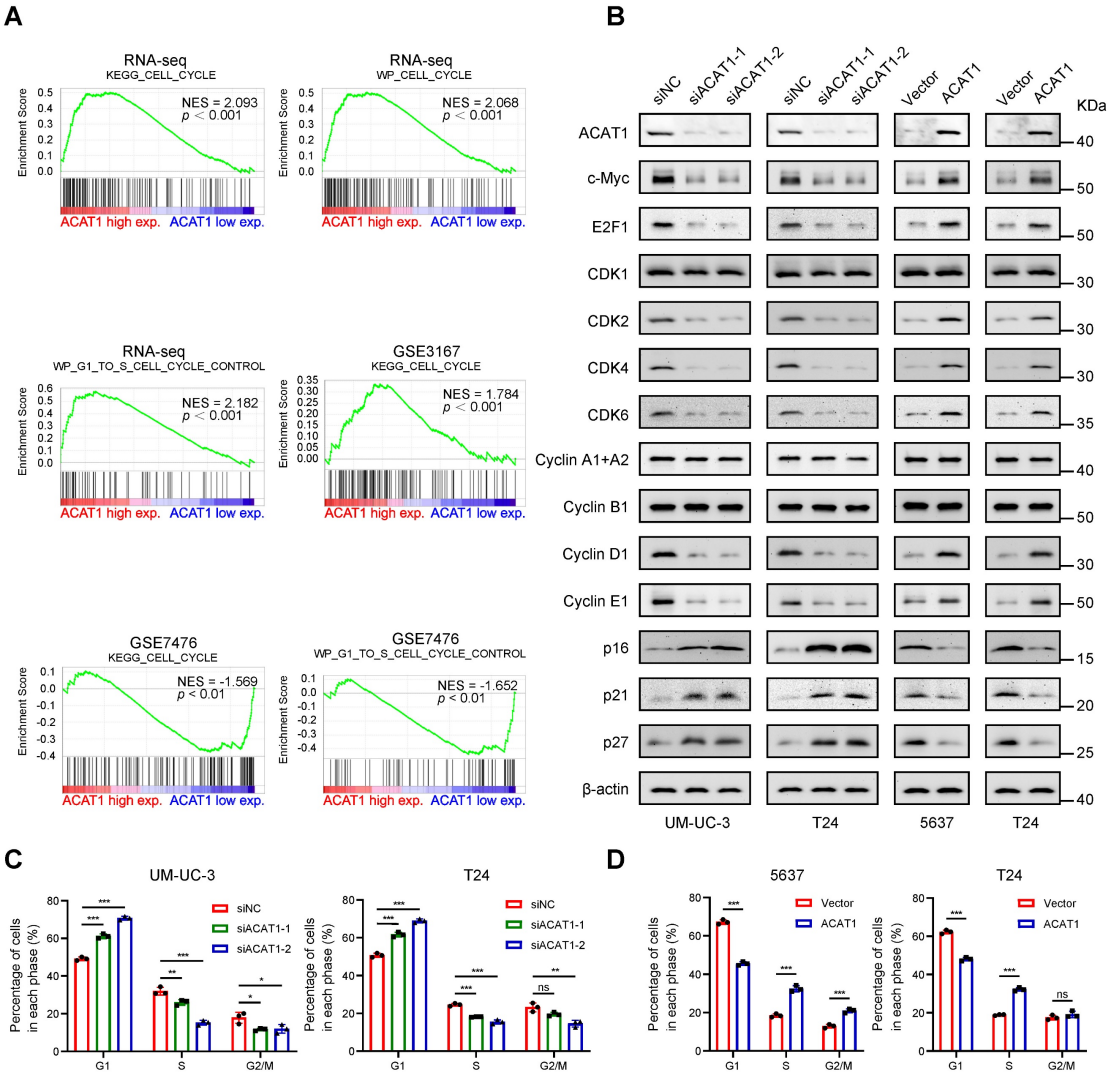
** Knockdown of *ACAT1* caused G1-phase cell cycle arrest in BLCA cells. (A)** GSEA of the RNA-seq data showed that the GSE3167 and GSE7476 datasets were enriched in the cell cycle and cell cycle control of the G1 to S phase. **(B)** Changes in the expression of cell cycle-related proteins after ACAT1 knockdown (UM-UC-3 and T24 cells) or overexpression (5637 and T24 cells) were detected by immunoblotting. **(C-D)** Statistical analysis of BLCA cells measured by flow cytometry after knockdown (UM-UC-3 and T24 cells) or overexpression (5637 and T24 cells) of ACAT1. ns: not significant; *: *p* < 0.05; **: *p* < 0.01; ***: *p* < 0.001*.*

GSEA revealed enrichment of the cell cycle and cell cycle control of the G1 to S phase in numerous GEO datasets (GSE3167 and GSE7476) and RNA-seq data (Fig. 4A). Immunoblotting demonstrated that knockdown of* ACAT1* decreased the expression of c-Myc, E2F1, CDK2, CDK4, CDK6, Cyclin D1, and Cyclin E1 while increasing the expression of p16, p21 and p27 (Fig. 4B). Conversely, ACAT1 overexpression had the opposite effect (Fig. 4B). Flow cytometry was used to assess the influence of ACAT1 interference on the cell cycle in BLCA cells. The results demonstrated that *ACAT1* knockdown increased the percentage of cells in G1 phase of UM-UC-3 and T24 cells, indicating G1 phase cell cycle arrest (Fig. 4C and Supplementary Fig. S4A, S4C). Conversely, ACAT1 overexpression reduced the percentage of G1-phase cells of 5637 and T24 cells, suggesting that the cell cycle was promoted (Fig. 4D and Supplementary Fig. S4B, S4D). In conclusion, we revealed that ACAT1 regulated the cell cycle in BLCA cells.

### Knockdown of *ACAT1* downregulated the AKT/GSK3β signaling pathway

**Figure 5:**
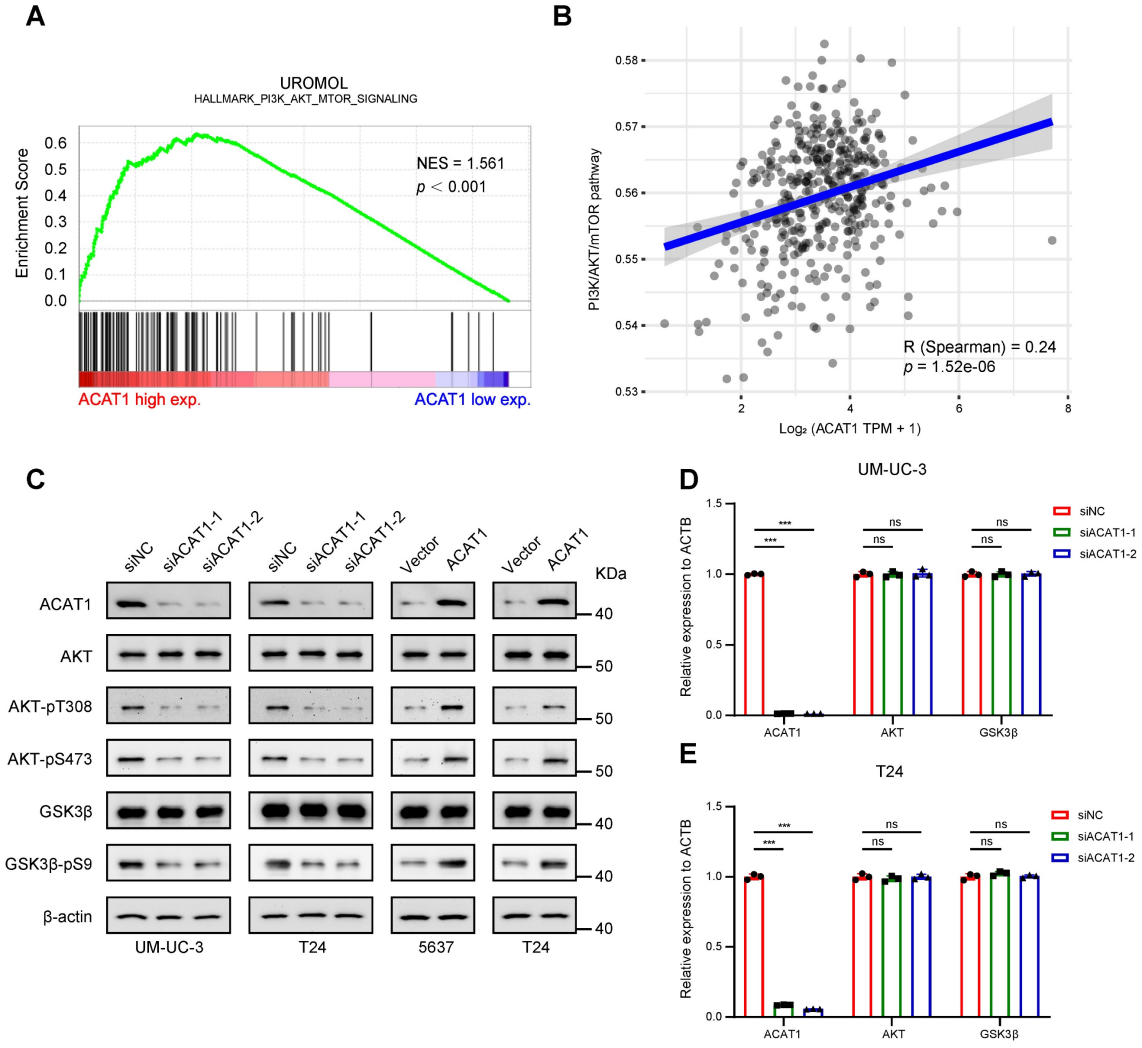
** Knockdown of *ACAT1* downregulated the AKT/GSK3β signaling pathway. (A)** GSEA of the UROMOL cohort data showed enrichment of the PI3K/AKT/mTOR signaling pathway.** (B)** Spearman correlation analysis between *ACAT1* and pathway scores. The ordinate represents the pathway score, and the abscissa represents gene expression. **(C)** Immunoblotting was performed to detect changes in the expression of AKT/GSK3β signaling pathway-related proteins after knockdown (UM-UC-3 and T24 cells) or overexpression (5637 and T24 cells) of ACAT1. **(D-E)** Statistical analysis of the changes in the AKT and GSK3β mRNAs detected by qRT-PCR after knockdown of *ACAT1* in UM-UC-3 and T24 cells, ns: not significant; *: *p* < 0.05; **: *p* < 0.01; ***: *p* < 0.001*.*

GSEA of the UROMOL cohort revealed enrichment of the PI3K/AKT/mTOR signaling pathway (Fig. 5A). GSVA was performed to determine the underlying mechanism, and we confirmed that *ACAT1* was positively correlated with the PI3K/AKT/mTOR signaling pathway (Fig. 5B). Subsequently, we explored the key proteins within this canonical signaling pathway. Western blotting demonstrated that *ACAT1* knockdown decreased AKT phosphorylation at T308 and S473 and reduced the phosphorylation of downstream GSK3β at S9 (Fig. 5C). Conversely, ACAT1 overexpression had the opposite effect (Fig. 5C). In addition, both total AKT protein and total GSK3β protein expression levels remained unchanged with ACAT1 knockdown or overexpression (Fig. 5C). Finally, we examined the transcriptional levels of AKT and GSK3β in this signaling pathway and determined that *ACAT1* knockdown had no effect on the transcriptional levels of these genes (Fig. 5D-E). Our results showed that ACAT1 regulated the AKT/GSK3β signaling pathway.

### ACAT1 promoted the proliferation and metastasis of BLCA cells through the AKT/GSK3β/c-Myc signaling pathway by modulating the cell cycle and EMT

**Figure 6:**
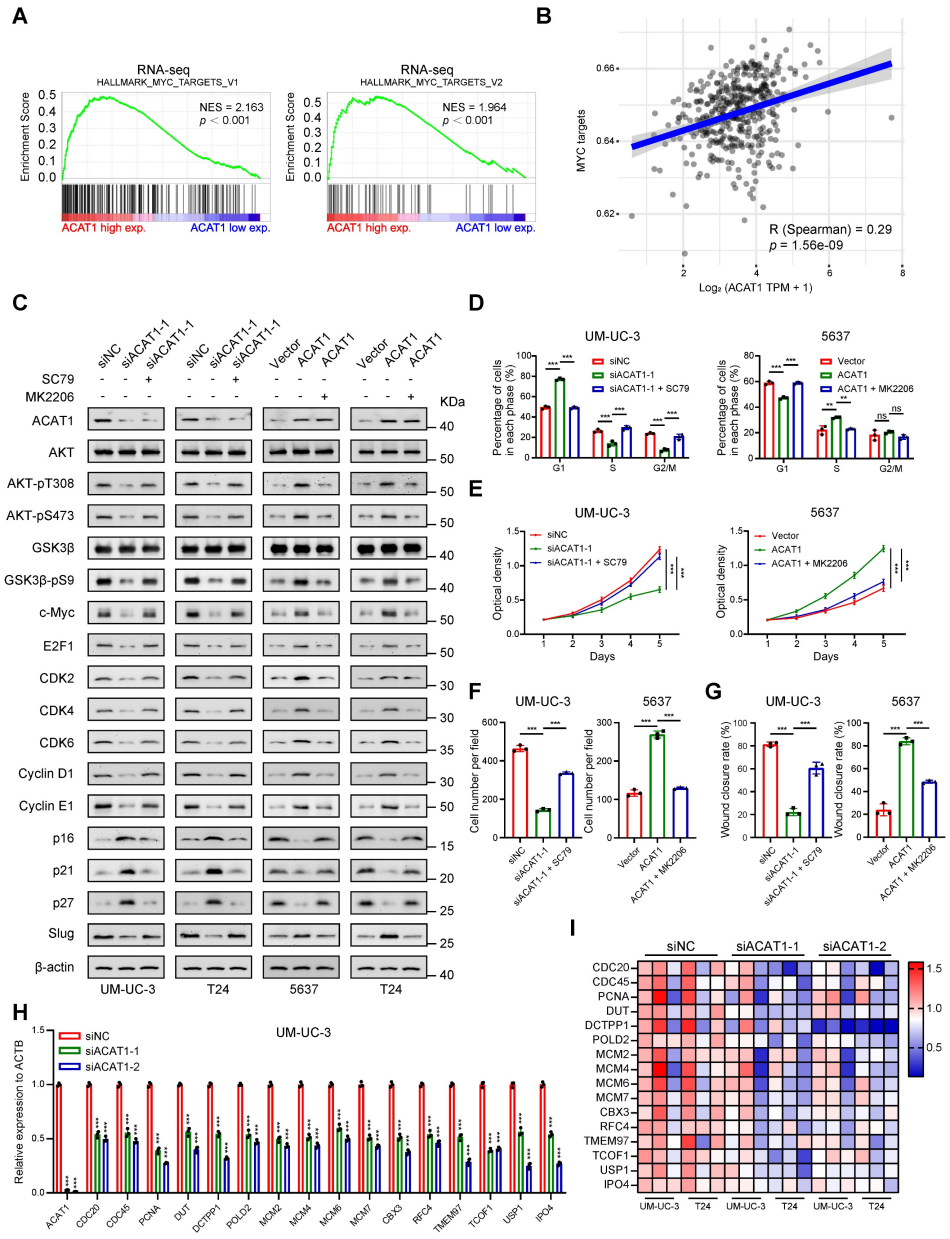
** ACAT1 promoted proliferation and metastasis of BLCA cells through AKT/GSK3β/c-Myc signaling pathway by modulating cell cycle and EMT. (A)** GSEA of RNA-seq data revealed enrichment of “MYC_TARGETS_V1” and “MYC_TARGETS_V2”.** (B)** Spearman correlation analysis between *ACAT1* and pathway scores. The ordinate represents the pathway score, and the abscissa represents gene expression. **(C)** Immunoblotting was performed to detect changes in the expression of proteins related to the AKT/GSK3β/c-Myc signaling pathway after treatment with SC79 (20 μM) or MK2206 (1 μM) following ACAT1 knockdown (UM-UC-3 and T24 cells) or overexpression (5637 and T24 cells), respectively. **(D)** Statistical analysis of BLCA cells detected by flow cytometry after treatment with SC79 (20 μM) or MK2206 (1 μM) following *ACAT1* knockdown in UM-UC-3 cells or overexpression in 5637 cells, respectively. **(E)** MTT experiments were performed on BLCA cells after SC79 (20 μM) or MK2206 (1 μM) treatment following ACAT1 knockdown in UM-UC-3 cells or overexpression in 5637 cells. **(F)** Statistical analysis of transwell assays performed on BLCA cells after using SC79 (20 μM) or MK2206 (1 μM) treatment following *ACAT1* knockdown in UM-UC-3 cells or overexpression in 5637 cells.** (G)** Statistical analysis of wound healing experiments performed on BLCA cells after SC79 (20 μM) or MK2206 (1 μM) treatment following *ACAT1* knockdown in UM-UC-3 cells or overexpression in 5637 cells.** (H)** Statistical analysis of changes in the mRNA levels of several downstream c-Myc molecules in UM-UC-3 cells after *ACAT1* knockdown was measured using qRT-PCR.** (I)** Heatmap showing the differences in the mRNA expression of several c-Myc target genes based on RNA-seq data. Red/blue represents high/low mRNA expression; ns: not significant; *: *p* < 0.05; **: *p* < 0.01; ***: *p* < 0.001*.*

Previous findings indicate that the depletion of *ACAT1* results in the inhibition of cell migration, cell cycle arrest at the G1 phase and the downregulation of the AKT/GSK3β signaling pathway, while ACAT1 overexpression has the opposite effects. This observation prompted the hypothesis that the AKT/GSK3β/c-Myc signaling pathway is intricately linked to the regulation of cell cycle and EMT. To explore this hypothesis, we conducted GSEA using RNA-seq data, which revealed enrichment of "MYC_TARGETS_V1" and "MYC_TARGETS_V2" (Fig. 6A). Furthermore, through GSVA, we found that *ACAT1* was positively correlated with MYC targets (Fig. 6B). Integrating these bioinformatics analyses with previous findings, we postulate that ACAT1 promotes BLCA cell proliferation and metastasis by modulating the cell cycle and EMT through the AKT/GSK3β/c-Myc signaling pathway.

To experimentally validate this hypothesis, we initially scrutinized the proteins involved in this signaling pathway. Western blotting revealed that knockdown of *ACAT1* suppressed the expression of AKT-pT308, AKT-pS473, GSK3β-pS9, c-Myc, E2F1, CDK2/4/6, Cyclin D1/E1, and Slug and increased p16/21/27. Notably, an AKT phosphorylation activator (SC79) reversed these effects (Fig. 6C). Conversely, ACAT1 overexpression elevated AKT-pT308, AKT-pS473, GSK3β-pS9, c-Myc, E2F1, CDK2/4/6, Cyclin D1/E1, and Slug and decreased p16/21/27; these effects were reversed by the AKT inhibitor (MK2206) (Fig. 6C). Subsequently, we investigated the impact of an AKT agonist or inhibitor on the cell cycle in the presence of ACAT1 interference by flow cytometry. *ACAT1* knockdown-induced G1 phase arrest was ameliorated by SC79 in UM-UC-3 and T24 cells, while ACAT1 overexpression-induced cell cycle arrest was counteracted by MK2206 in 5637 and T24 cells (Fig. 6D and Supplementary Fig. S4E-I). MTT and colony formation assays further revealed that the decrease resulting from *ACAT1* knockdown could be rescued by SC79 in UM-UC-3 and T24 cells, whereas the increase in cell growth due to ACAT1 overexpression could be attenuated by MK2206 in 5637 and T24 cells (Fig. 6E and Supplementary Fig. S4J, S5A-D). Transwell and wound healing assays also demonstrated that the decrease in cell migration caused by *ACAT1* knockdown could be reversed by SC79 in UM-UC-3 and T24 cells, while the increase in cell migration caused by ACAT1 overexpression could be attenuated by MK2206 in both 5637 and T24 cells (Fig. 6F-G and Supplementary Fig. S5E-L).

Finally, utilizing the gene sets corresponding to "MYC_TARGETS_V1" and "MYC_TARGETS_V2" obtained from GSEA, we generated an RNA-seq heatmap. The results demonstrated that in BLCA cells following *ACAT1* knockdown, several downstream molecules of c-Myc were downregulated to varying degrees (Fig. 6I). qRT-PCR confirmed the transcriptional downregulation of multiple c-Myc downstream molecules, including CDC20, CDC45, MCM2, MCM4, MCM6, and MCM7, after *ACAT1* knockdown in UM-UC-3 and T24 cells (Fig. 6H and Supplementary Fig. S5N).

Taken together, these findings support the assertion that ACAT1 promotes BLCA cell proliferation and metastasis through the AKT/GSK3β/c-Myc signaling pathway by modulating the cell cycle and EMT.

## Discussion

Previous investigations have extensively explored the roles of ACAT1 in various malignancies 19-28, yet its function in BLCA has largely not been explored. Among our 9 pairs of BLCA tissues and paired adjacent tissues obtained during surgery, we observed elevated mRNA expression of *ACAT1* in the BLCA tissues. Analysis of clinical data from BLCA patients in the TCGA database and HBlaU079Su01 cohort revealed a positive association between poor prognosis and high ACAT1 expression, consistent with findings in prostate and uterine cancers 23, 26, 27. Drawing from previous research indicating that ACAT1 promotes proliferation and metastasis in cancers such as prostate and uterine cancer 23-27, we employed bioinformatics to establish an association between *ACAT1* and both cell proliferation and EMT. Subsequent *in vitro* and *in vivo* experiments demonstrated that *ACAT1* knockdown impeded BLCA cell migration and proliferation, resulting in diminished distant lung metastasis and tumor growth. Further assessment of subcutaneous tumor tissues from nude mice revealed decreased expression of the cell proliferation marker Ki67 in the *ACAT1* stable knockdown group, confirming the role of *ACAT1* as an oncogene in BLCA development.

Cell cycle dysregulation is a known contributor to uncontrolled cell proliferation and cancer progression 29, 30. Abnormalities in cell cycle regulatory proteins, such as cyclins, cyclin-dependent kinases (CDKs) and cyclin-dependent kinase inhibitors, are implicated in this process 31. In various malignancies, such as BLCA, an abnormal increase in CDKs (including CDK2/4/6) and cyclins (including Cyclin D1/E1) and a decrease in CDK inhibitors (including p16/21/27) are common 32-37. Elevated levels of E2F1, an E2F transcription factor family member, are also associated with dysregulated G1/S cell cycle transition and cancer progression 38, 39. The multifunctional transcription factor c-Myc has been implicated in promoting G1/S cell cycle transition and cell cycle progression 32. The GSEA results obtained from our own RNA-seq data and from external GEO datasets supported the enrichment of cell cycle-related processes. Western blotting and flow cytometry demonstrated that ACAT1 knockdown or overexpression can affect the cell cycle, consistent with the findings of previous studies. Notably, CDK1 and Cyclin A/B expression remained largely unaffected, suggesting a predominant role in G2/M phase regulation 32, 40.

As a canonical signaling pathway, the PI3K/AKT/mTOR pathway plays a crucial role in cell biology, especially in the regulation of cell proliferation and the cell cycle 41. In breast cancer, acetylation can increase AKT phosphorylation indirectly and ultimately increase AKT signaling activity 42. Inspired by this evidence, we hypothesized that ACAT1 may be correlated with the PI3K/AKT/mTOR signaling pathway. Our bioinformatics analyses revealed a positive association between *ACAT1* and the PI3K/AKT/mTOR signaling pathway. Western blot analysis demonstrated that *ACAT1* knockdown decreased the expression of AKT-pT308, AKT-pT473, and GSK3β-pS9 in this pathway, while ACAT1 overexpression had the opposite effect. Therefore, we suggest that ACAT1 regulates the AKT/GSK3β signaling pathway in BLCA. While interference with ACAT1 expression impacts AKT phosphorylation, ACAT1, an acetyltransferase, lacks intrinsic kinase activity. *ACAT1* knockdown did not alter the total AKT protein at the transcriptional or translational level. Therefore, further exploration is warranted. First, considering the reported interactions between SIRT7, FKBP51, and AKT in breast cancer 42, we hypothesize that ACAT1 may regulate the activity of AKT by acetylating a certain kinase and regulating its ability to phosphorylate AKT. Second, given that SIRT1 enhances AKT deacetylation 43, we propose that ACAT1 may directly acetylate AKT, thereby increasing AKT phosphorylation.

The role of AKT in regulating c-Myc through a GSK3β-dependent mechanism has been highlighted by multiple studies. AKT phosphorylates S9 to inactivate GSK3β, subsequently phosphorylating T58 to reduce c-Myc degradation through the proteasome pathway 44-48. Combined with previous bioinformatic analyses and experimental results, we suggest that ACAT1 modulates the AKT/GSK3β/c-Myc signaling pathway in BLCA. Given the diverse functions of c-Myc, including cycle regulation and cell proliferation, it is associated with 20% of human cancers, and its promotion of the cell cycle in carcinoma cells induces the expression of CDKs, cyclins and the E2F family and inhibits CDKs inhibitors 32, 49. Therefore, we propose that through the AKT/GSK3β/c-Myc signaling pathway, ACAT1 promotes BLCA cell proliferation by modulating the cell cycle. In addition, it has been revealed that c-Myc plays a role in the regulation of EMT-related transcription factors such as Snail and Slug, thus participating in tumor metastasis 50. The c-Myc/Snail axis has also been reported to regulate EMT in BLCA 51. Thus, we speculate that through the AKT/GSK3β/c-Myc signaling pathway, ACAT1 promotes BLCA cell metastasis by regulating EMT.

Supporting evidence from both bioinformatics analyses and experimental studies reinforced this hypothesis. Enrichment analyses of RNA-seq and TCGA data highlighted the enrichment of MYC targets. Western blotting revealed that the changes in the expression of AKT-pT308, AKT-pS473, GSK3β-pS9, c-Myc, and c-Myc-regulated proteins caused by *ACAT1* knockdown or overexpression can be reversed by the AKT phosphorylation agonist SC79 or the AKT phosphorylation inhibitor MK2206. Flow cytometry, MTT and colony formation assays as well as transwell and wound healing assays also demonstrated that the cell cycle regulation, cell proliferation and migration affected by *ACAT1* knockdown or overexpression can be reversed by SC79 or MK2206. RNA-seq heatmap analysis and qRT-PCR validation confirmed the downregulation of c-Myc downstream molecules in *ACAT1* knockdown BLCA cells, linking these molecules to cell cycle regulation and proliferation in various malignancies, including glioblastoma 52, 53, nasopharyngeal carcinoma 54, esophageal cancer 55, breast cancer 56, 57, cervical cancer 58, lung cancer 59, liver cancer 60-62, bladder cancer 63, prostate cancer 64, acute myeloid leukemia 65 and diffuse large B-cell lymphoma 66. In addition, reports on c-Myc-regulated downstream molecules involved in EMT, such as POLD2 52, TMEM97 56, DCTPP1 64, MCM6 61 and CBX3 53, have been published. Moreover, these molecules were also downregulated at the transcriptional level according to our qRT-PCR validation. The above evidence confirmed our view that ACAT1 promoted the proliferation and metastasis of BLCA cells by modulating the cell cycle and EMT through the AKT/GSK3β/c-Myc signaling pathway.

The acetylation capacity of mitochondrion-localized acetyltransferases was reported to be correlated with autophagy, apoptosis and oxidative stress 67-69. In prostate cancer, ACAT1 inhibited autophagy or oxidative stress by preventing FUS from transcribing LC3B or scavenging ROS, thereby promoting tumorigenesis 67. Inspired by these findings, we explored whether apoptosis, autophagy and oxidative stress are involved in the tumorigenesis of BLCA by ACAT1 in subsequent studies. Additionally, due to the limited number of surgical specimens collected, it is not rigorous to solely evaluate the disparity in ACAT1 expression between cancerous and adjacent tissues based on mRNA expression levels from these 9 pairs of bladder tissues. To enhance the reliability of our analysis results, we will continue to accumulate surgical specimens in future studies. Furthermore, since these pathological samples were obtained from surgeries at the initial stages of cancer, accurately assessing the discrepancy in ACAT1 expression levels between BLCA tissues and paired adjacent tissues during tumor metastasis has become challenging. To address this issue, we plan to expand our patient cohort by including individuals with diverse pathological grades and stages.

In conclusion, this study identified *ACAT1* as an oncogene and a potential biomarker in BLCA. ACAT1 promoted the proliferation of BLCA cells through the AKT/GSK3β/c-Myc signaling pathway by modulating the cell cycle (Supplementary Fig. S5M, left panel). Additionally, ACAT1 regulates EMT and enhances the metastatic potential of BLCA cells via the AKT/GSK3β/c-Myc signaling pathway (Supplementary Fig. S5M, right panel).

## Supplementary Material

Supplementary figures and tables.

## Abbreviations

ACAT1: acetyl-CoA acetyltransferase 1
AKT: protein kinase B
BLCA: bladder cancer
CCLE: Cancer Cell Line Encyclopedia
CDK: cyclin dependent kinase
CI: confidence interval
EMA: European Medicines Agency
EMT: epithelial-mesenchymal transition
FDA: Food and Drug Administration
GEO: Gene Expression Omnibus
GSEA: gene set enrichment analysis
GSK3β: glycogen synthase kinase 3β
GSVA: gene set variation analysis
H&E: hematoxylin & eosin
HR: hazard ratio
IHC: immunohistochemistry
MMP9: matrix metalloproteinase 9
mTOR: mammalian target of rapamycin
NCBI: National Center of Biotechnology Information
NES: normalized enrichment score
ns: not significant
OR: odds ratio
ORR: overall response rate
OS: overall survival
PCR: polymerase chain reaction
PFS: progression free survival
PI3K: phosphatidylinositol 3-kinase
qRT-PCR: quantitative reverse transcription PCR
SD: standard deviation
SPF: specific pathogen-free
TCGA: The Cancer Genome Atlas
