# Supplementary material for: ACAT1 promotes proliferation and metastasis of bladder cancer via AKT/GSK3β/c-Myc signaling pathway
Source: J Cancer. 2024 Apr 23;15(11):3297–312. doi: 10.7150/jca.95549 (PMC11134450; doi:10.7150/jca.95549)
Supplement: Supplementary file 1 — Supplementary figures and tables. [file jcav15p3297s1.pdf]

Supplementary Information

ACAT1 promotes proliferation and metastasis of bladder cancer via AKT/GSK3β/c-Myc signaling pathway

Supplementary Figures: Pages 2-9

Supplementary Tables: Pages 10-15

Supplementary Figures S1-S5

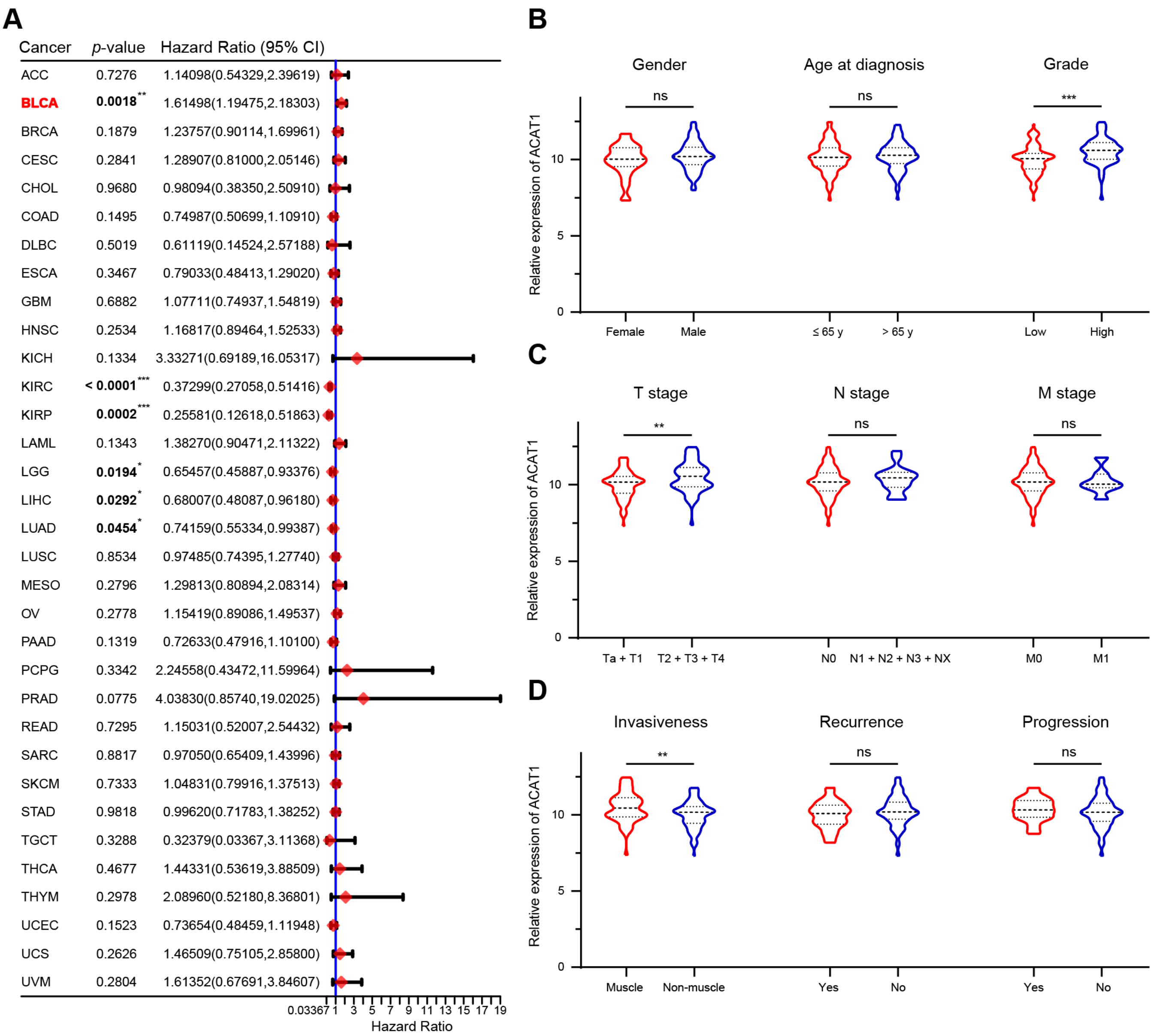

Supplementary Figure S1. ACAT1 indicated poor prognosis in BLCA patients.

(A) Forest plot showing the results of univariate Cox survival analyses of *ACAT1* in multiple tumors, with *p*-values, hazard ratios (HRs), and 95% confidence intervals (CIs). **(B-D)** Differences in *ACAT1* mRNA expression levels in patients of different sexes, ages at diagnosis, grades, T stages, N stages, M stages and patients with or without muscle invasion, recurrence, and progression in GSE13507 dataset, ns: not significant, *: $p < 0.05$, **: $p < 0.01$, ***: $p < 0.001$.

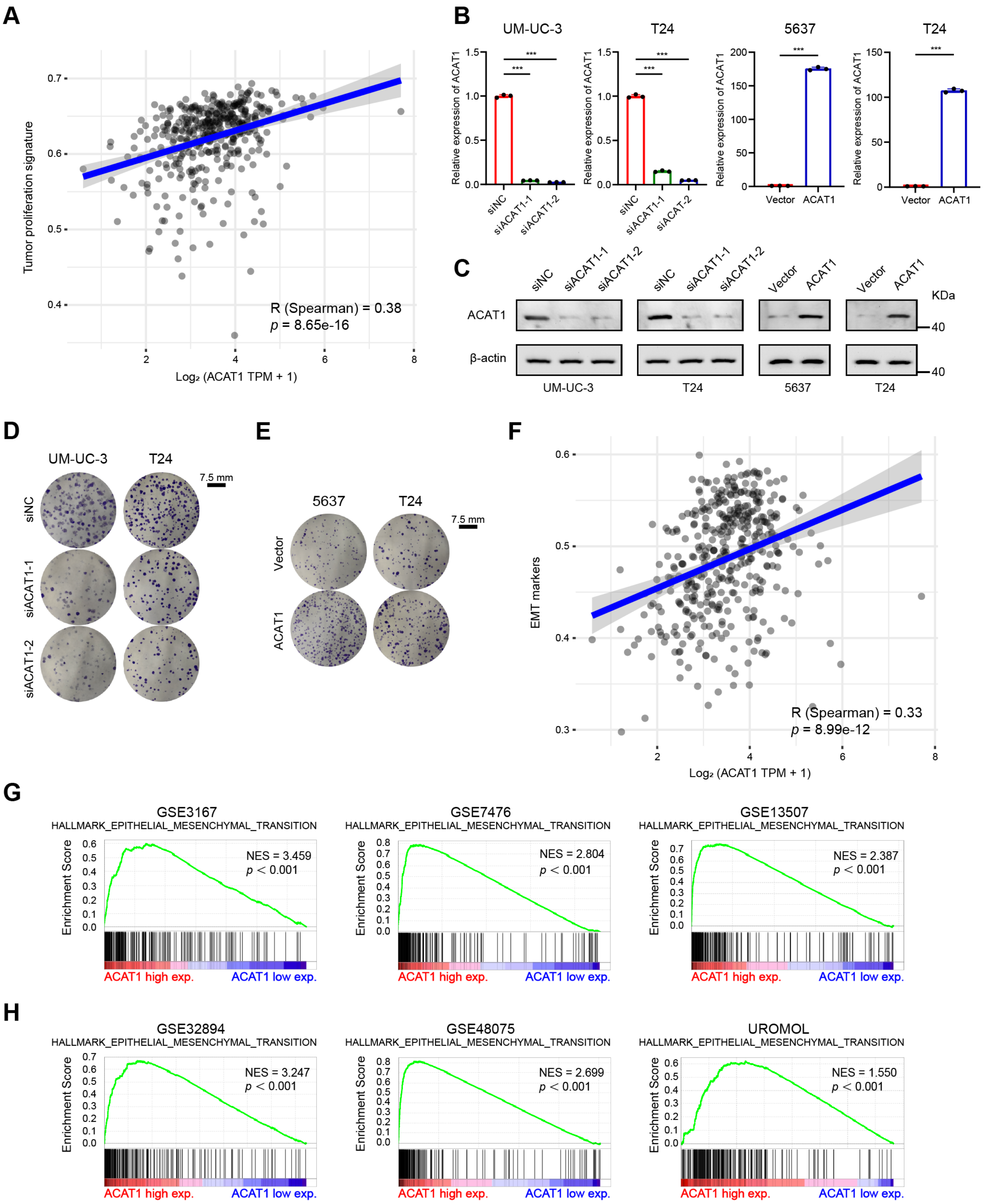

Supplementary Figure S2. ACAT1 regulated the proliferation and metastasis of BLCA cells.

(A) Spearman correlation analysis between *ACAT1* and pathway scores. The abscissa represents gene expression, and the ordinate represents the pathway score. **(B-C)** The knockdown efficiency in UM-UC-3 and T24 cells and overexpression efficiency in 5637 and T24 cells of siRNAs and plasmids in BLCA cell lines

were verified by qRT-PCR and Western blot. **(D-E)** Colony formation assays were performed on BLCA cells after knockdown (UM-UC-3 and T24 cells) or overexpression (5637 and T24 cells) of ACAT1 (n = 3). The scale bar is 7.5 mm. **(F)** Spearman correlation analysis between *ACAT1* and pathway scores. The abscissa represents gene expression, and the ordinate represents the pathway score. **(G-H)** GSEA of GSE3167, GSE7476, GSE13507, GSE32894, and GSE48075 datasets and UROMOL cohort data revealed enrichment of genes related to EMT, ns: not significant, *: $p < 0.05$, **: $p < 0.01$, ***: $p < 0.001$.

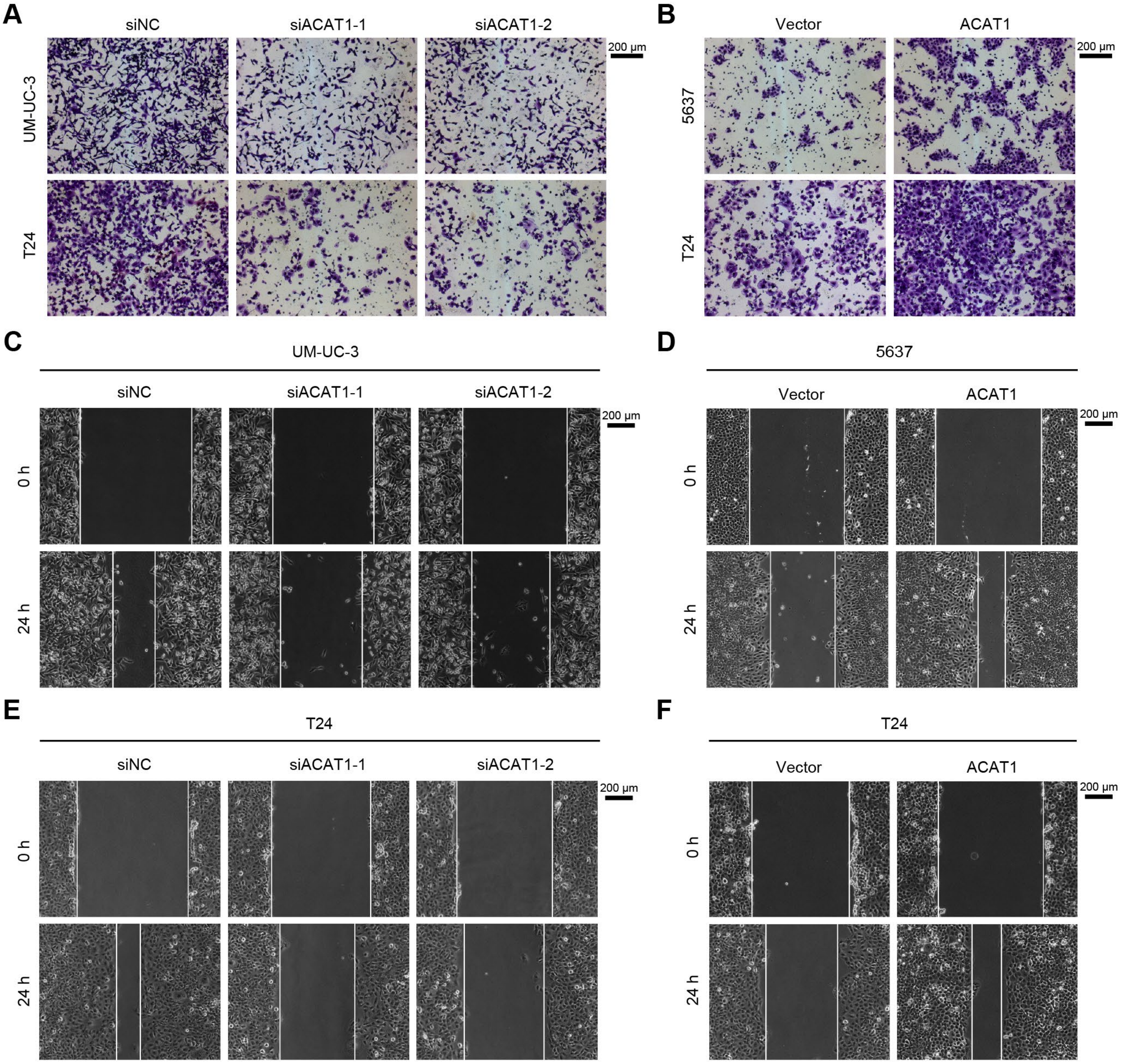

Supplementary Figure S3. ACAT1 regulated the metastasis of BLCA cells.

(A-B) Transwell assays were performed on BLCA cells after knockdown (UM-UC-3 and T24 cells) or overexpression (5637 and T24 cells) of ACAT1 (n = 3). The scale bar is 200 μm. **(C-F)** Wound healing assays were performed on BLCA cells after knockdown (UM-UC-3 and T24 cells) or overexpression (5637 and T24 cells) of ACAT1 (n = 3). The scale bar is 200 μm.

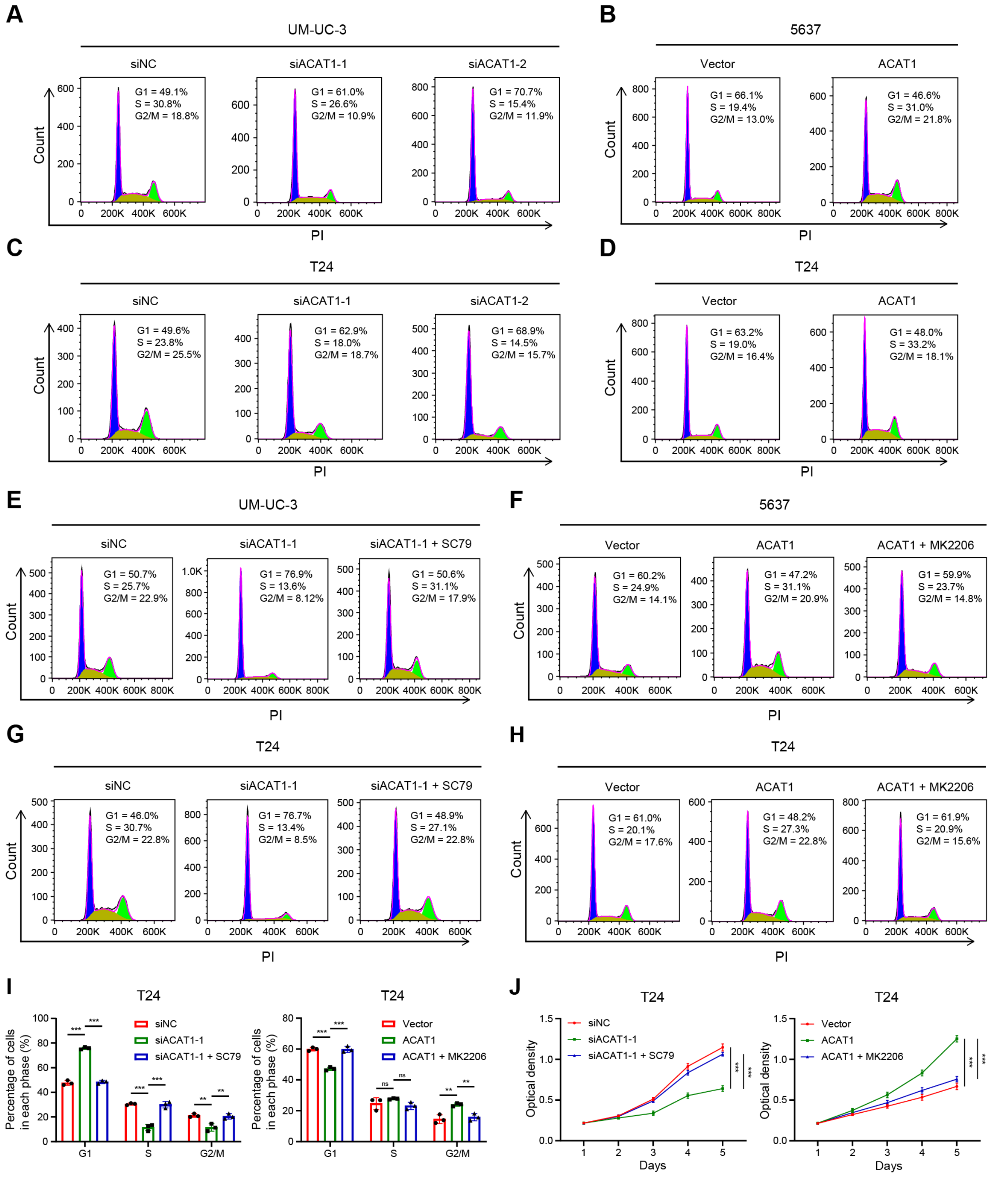

Supplementary Figure S4. ACAT1 affected the cell cycle of BLCA cells.

(A-D) Flow cytometry of BLCA cells after knockdown (UM-UC-3 and T24 cells) or overexpression (5637 and T24 cells) of ACAT1 (n = 3). **(E-H)** Flow cytometry of BLCA cells after treatment with SC79 (20 μM) or MK2206 (1 μM) following ACAT1 knockdown (UM-UC-3 and T24 cells) or overexpression (5637 and T24

cells) (n = 3). **(I)** Statistical analysis of BLCA cells detected by flow cytometry after treatment with SC79 (20 μM) or MK2206 (1 μM) following ACAT1 knockdown or overexpression in T24 cells. **(J)** MTT assays were performed on BLCA cells after SC79 (20 μM) or MK2206 (1 μM) treatment following ACAT1 knockdown or overexpression in T24 cells.

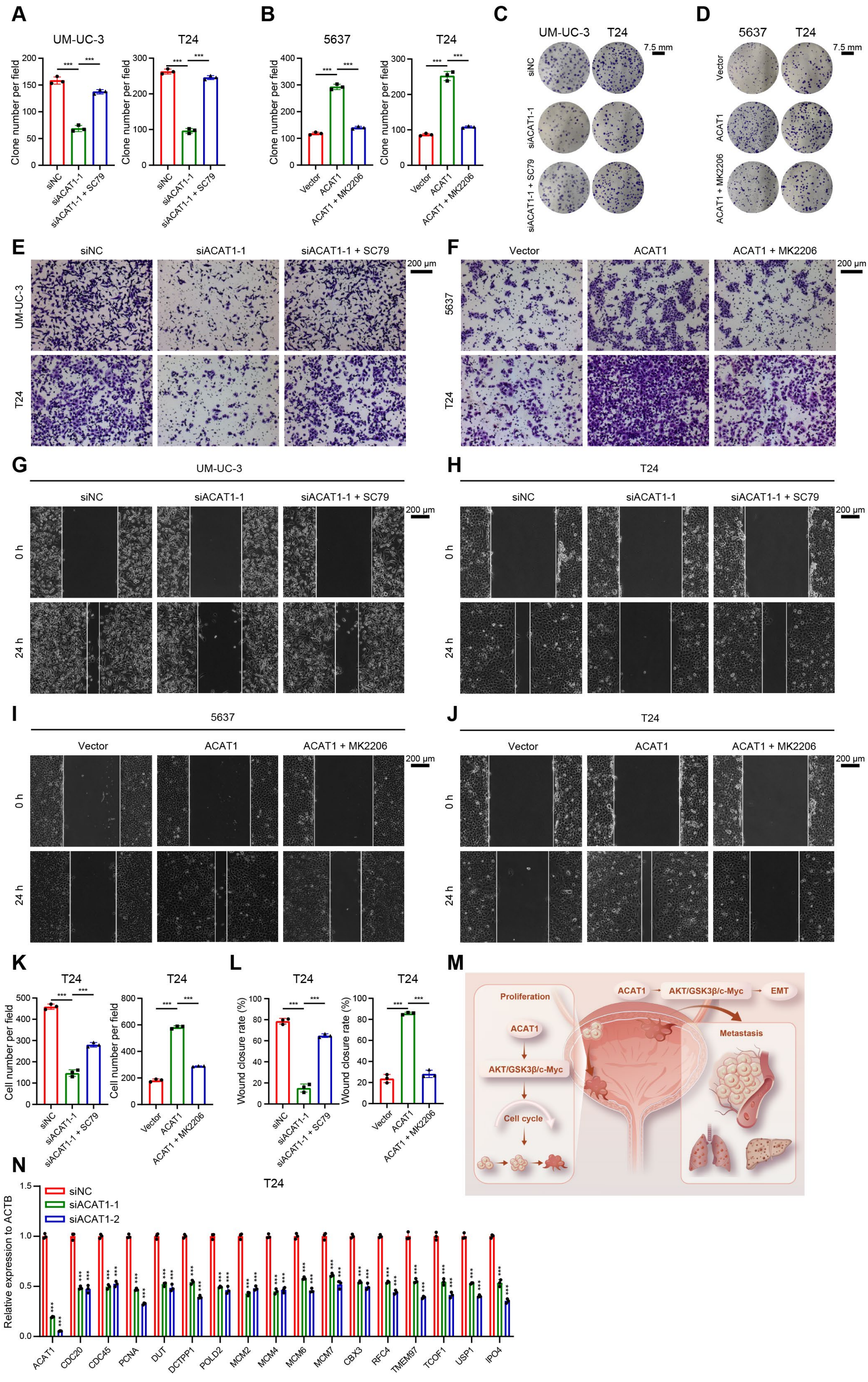
A
UM-UC-3
T24
Clone number per field
siNC
siACAT1-1
siACAT1-1 + SC79
B
5637
T24
Vector
ACAT1
ACAT1 + MK2206
C
UM-UC-3
T24
7.5 mm
D
5637
T24
E
siNC
siACAT1-1
siACAT1-1 + SC79
200 μm
F
Vector
ACAT1
ACAT1 + MK2206
G
UM-UC-3
0 h
24 h
H
T24
I
5637
J
T24
K
Cell number per field
L
Wound closure rate (%)
M
Proliferation
ACAT1
AKT/GSK3β/c-Myc
Cell cycle
EMT
Metastasis
N
T24
Relative expression to ACTB
siACAT1-2
ACAT1
CDC20
CDC45
PCNA
DUT
DCTPP1
POLD2
MCM2
MCM4
MCM6
MCM7
CBX3
RFC4
TMEM97
TCOF1
USP1
IPO4

Supplementary Figure S5. ACAT1 promoted the proliferation and metastasis of BLCA cells through AKT/GSK3β/c-Myc signaling pathway by modulating the cell cycle and EMT.

(A-B) Statistical analysis of colony formation assays performed on BLCA cells after treatment with SC79 (20 μM) or MK2206 (1 μM) following ACAT1 knockdown (UM-UC-3 and T24 cells) or overexpression (5637 and T24 cells). **(C-D)** Colony formation assays were performed on BLCA cells after SC79 (20 μM) or MK2206 (1 μM) treatment following ACAT1 knockdown (UM-UC-3 and T24 cells) or overexpression (5637 and T24 cells) (n = 3). The scale bar is 7.5 mm. **(E-F)** Transwell assays were performed on BLCA cells after SC79 (20 μM) or MK2206 (1 μM) treatment following ACAT1 knockdown (UM-UC-3 and T24 cells) or overexpression (5637 and T24 cells) (n = 3). The scale bar is 200 μm. **(G-J)** Wound healing experiments were performed on BLCA cells after SC79 (20 μM) or MK2206 (1 μM) treatment following ACAT1 knockdown (UM-UC-3 and T24 cells) or overexpression (5637 and T24 cells) (n = 3). The scale bar is 200 μm. **(K)** Statistical analysis of transwell assays performed on BLCA cells after treatment with SC79 (20 μM) or MK2206 (1 μM) following ACAT1 knockdown or overexpression in T24 cells. **(L)** Statistical analysis of wound healing experiments performed on BLCA cells after treatment with SC79 (20 μM) or MK2206 (1 μM) following ACAT1 knockdown or overexpression in T24 cells. **(M)** Mechanistic diagram of ACAT1 regulation in BLCA. Left panel: ACAT1 promoted BLCA cells proliferation by modulating the cell cycle through the AKT/GSK3β/c-Myc signaling pathway. Right panel: ACAT1 regulated the EMT process and promoted the metastatic ability of BLCA cells via the AKT/GSK3β/c-Myc signaling pathway. **(N)** Statistical analysis of the changes in the mRNA levels of c-Myc target genes measured by qRT-PCR in T24 cells after *ACAT1* knockdown, ns: not significant, *: $p < 0.05$, **: $p < 0.01$, ***: $p < 0.001$.

Supplementary Tables S1-S6

Supplementary Table S1. Cox analyses of cancer-specific survival among BLCA patients from TCGA.

BLCA patients (n = 371)	Univariate analyses			Multivariate analyses		
	HR	95% CI	*p*-value	HR	95% CI	*p*-value
***ACAT1* expression** (Low vs. High)	1.891	1.346 - 2.658	**< 0.001*****	1.736	1.225 - 2.458	**0.002****
**Gender** (Female vs. Male)	0.867	0.606 - 1.242	0.436	-	-	-
**Age at diagnosis** (≤ 65 y vs. > 65 y)	1.891	1.305 - 2.739	**0.001****	1.929	1.330 - 2.798	**0.001****
**Grade** (Low vs. High)	21.740	0.253 - 1869.996	0.175	-	-	-
**Stage** (I + II vs. III + IV)	2.922	1.802 - 4.737	**< 0.001*****	2.189	1.325 - 3.615	**0.002****
**T stage** (T0 + T1 vs. T2 + T3 + T4)	20.666	0.066 - 6446.135	0.301	-	-	-
**N stage** (N0 vs. N1 + N2 + N3 + NX)	2.313	1.658 - 3.227	**< 0.001*****	1.900	1.344 - 2.686	**< 0.001*****
**M stage** (M0 vs. M1 + MX)	1.541	1.105 - 2.149	**0.011***	1.237	0.875 - 1.749	0.229

Abbreviations: CI, Confidence Interval; HR, Hazard Ratio.

Supplementary Table S2. Clinicopathological statistics of BLCA patients from TCGA based on *ACAT1* expression level.

Clinicopathological Features	*ACAT1* mRNA expression level		Total	OR	95% CI	*p*-value
	Low	High				
**Overall**	186	186	372			
**Gender**						
Female	46 (24.73%)	51 (27.42%)	97	0.870	0.553 - 1.399	0.6368
Male	140 (75.27%)	135 (72.58%)	275			
**Age at diagnosis**						
≤ 65 y	77 (41.40%)	64 (34.41%)	141	1.347	0.892 - 2.046	0.1996
> 65 y	109 (58.60%)	122 (65.59%)	231			
**Grade**						
Low	19 (10.22%)	1 (0.54%)	20	21.050	3.714 - 221.100	**< 0.0001*****
High	167 (89.78%)	185 (99.46%)	352			
**Stage**						
I + II	64 (34.41%)	42 (22.58%)	106	1.799	1.141 - 2.820	**0.0156***
III + IV	122 (65.59%)	144 (77.42%)	266			
**T stage**						
T0 + T1	3 (1.61%)	1 (0.54%)	4	3.033	0.448 - 39.580	0.6230
T2 + T3 + T4	183 (98.39%)	185 (99.46%)	368			
**N stage**						
N0	117 (62.90%)	105 (56.45%)	222	1.308	0.855 - 1.966	0.2449
N1 + N2 + N3 + NX	69 (37.10%)	81 (43.55%)	150			
**M stage**						
M0	111 (59.68%)	69 (37.10%)	180	2.510	1.643 - 3.808	**< 0.0001*****
M1 + MX	75 (40.32%)	117 (62.90%)	192			

Abbreviations: CI, Confidence Interval; OR, Odds Ratio.

Supplementary Table S3. Clinicopathological statistics of BLCA patients from GSE13507 based on *ACAT1* expression level.

Clinicopathological Features	*ACAT1* mRNA expression level		Total	OR	95% CI	*p*-value
	Low	High				
**Overall**	80	85	165			
**Gender**						
Female	16 (20.00%)	14 (16.47%)	30	1.268	0.582 - 2.736	0.6868
Male	64 (80.00%)	71 (83.53%)	135			
**Age at diagnosis**						
≤ 65 y	39 (48.75%)	35 (41.18%)	74	1.359	0.718 - 2.472	0.3508
> 65 y	41 (51.25%)	50 (58.82%)	91			
**Grade**						
Low	61 (76.25%)	44 (51.76%)	105	2.992	1.538 - 5.923	**0.0012****
High	19 (23.75%)	41 (48.24%)	60			
**Invasiveness**						
Muscle invasive	27 (33.75%)	35 (41.18%)	62	0.728	0.383 - 1.354	0.3395
Non-muscle invasive	53 (66.25%)	50 (58.82%)	103			
**T stage**						
Ta + T1	53 (66.25%)	51 (60.00%)	104	1.309	0.701 - 2.490	0.4243
T2 + T3 + T4	27 (33.75%)	34 (40.00%)	61			
**N stage**						
N0	74 (92.50%)	75 (88.24%)	149	1.644	0.615 - 4.782	0.4347
N1 + N2 + N3 + NX	6 (7.50%)	10 (11.76%)	16			
**M stage**						
M0	76 (95.00%)	82 (96.47%)	158	0.695	0.171 - 2.664	0.7135
M1 + MX	4 (5.00%)	3 (3.53%)	7			
**Recurrence**						
Yes	19 (23.75%)	17 (20.00%)	36	1.246	0.608 - 2.605	0.5774
No	61 (76.25%)	68 (80.00%)	129			
**Progression**						
Yes	12 (15.00%)	19 (22.35%)	31	0.613	0.269 - 1.368	0.2396
No	68 (85.00%)	66 (77.65%)	134			

Abbreviations: CI, Confidence Interval; OR, Odds Ratio.

Supplementary Table S4. Sequences of siRNAs and lentivirus used in this study.

Target	Sequence (5′-3′)
siNC	UUCUCCGAACGUGUCACGUTT
siACAT1-1	GAGGCUCAAUGUUACACCATT
siACAT1-2	GUCUGGUUGUACUAGCAAATT
LV-shNC	TTCTCCGAACGTGTCACGT
LV-shACAT1	GTCTGGTTGTACTAGCAAA

Supplementary Table S5. Primers for qRT-PCR used in this study.

Gene	Forward primer (5′-3′)	Reverse primer (5′-3′)
ACAT1	GGAGGCTGGTGCAGGAAATA	AGCAAGGAAAGGCTGCCTAA
AKT	CTCTTTCCAGACCCACGACC	ACAGGTGGAAGAACAGCTCG
GSK3β	CTCAGGAGTGCGGGTCTTC	TGTTAGTCGGGCAGTTGGTG
CDC20	GCTTTGAACCTGAACGGTTTTG	TCTGGCGCATTTTGTGGTTTT
CDC45	CTTGAAGTTCCCGCCTATGAAG	GCATGGTTTGCTCCACTATCTC
PCNA	GCGTGAACCTCACCAGTATGT	TCTTCGGCCCTTAGTGTAATGAT
DUT	GGTGATCGAATTGCACAGCTC	TGAACCCCTTTCGGTGTCATC
DCTPP1	CGCCTCCATGCTGAGTTTG	CCAGGTTCCCCATCGGTTTTC
POLD2	CCATCAGCCAACAATGCCAC	CTAGCCGGAAGGGTTGTGA
MCM2	CCGTGACCTTCCACCATTTGA	GGTAGTCCCTTTCCATGCCAT
MCM4	CACCACACACAGTTATCCTGTT	CGAATAGGCACAGCTCGATAGAT
MCM6	TCGGGCCTTGAAAACATTCGT	TGTGTCTGGTAGGCAGGTCTT
MCM7	CCTACCAGCCGATCCAGTCT	CCTCCTGAGCGGTTGGTTT
CBX3	TAGATCGACGTGTAGTGAATGGG	TGTCTGTGGCACCAATTATTCTT
RFC4	CCGCTGACCAAGGATCGAG	AGGGAACGGGTTTGGCTTTC
TMEM97	ACACCATGACAACCTTAATTCCG	GGGCTCCGCAACATGAAAA
TCOF1	CGGGAGCTACTTCCCCTGAT	CAGAAGGGTTACGGGCTGAG
USP1	TCATTTCGGTTGAACAGCTCC	CCCTCAGTGTGTTAAGCAGTC
IPO4	CTCTGCGACCAGGTAGACGA	CCCATCAGACCCGATAAGGC
β-actin	GAGCACAGAGCCTCGCCTTT	TCATCATCCATGGTGAGCTGG

Supplementary Table S6. Antibodies used in this study.

Antigens	Host	IHC	WB	Supplier
ACAT1	Rabbit	1:100	1:1000	Proteintech, 16215-1-AP
N-cadherin	Rabbit		1:1000	CST, 13116
E-cadherin	Rabbit		1:1000	CST, 3195
Snail	Rabbit		1:1000	CST, 3879
Slug	Rabbit		1:1000	CST, 9585
MMP9	Rabbit		1:1000	Proteintech, 10375-2-AP
Vimentin	Rabbit		1:1000	CST, 5741
c-Myc	Rabbit		1:1000	CST, 18583
E2F1	Rabbit		1:1000	CST, 3742
CDK1	Mouse		1:1000	Abcam, ab18
CDK2	Rabbit		1:1000	Abcam, ab32147
CDK4	Rabbit		1:1000	Abcam, ab108357
CDK6	Rabbit		1:1000	CST, 13331
Cyclin A1+A2	Rabbit		1:1000	Abcam, ab185619
Cyclin B1	Rabbit		1:1000	Abcam, ab32053
Cyclin D1	Rabbit		1:1000	Abcam, ab134175
Cyclin E1	Rabbit		1:1000	Abcam, ab33911
p16	Rabbit		1:1000	Abcam, ab108349
p21	Rabbit		1:1000	CST, 2947
p27	Rabbit		1:1000	Proteintech, 25614-1-AP
AKT	Rabbit		1:1000	CST, 4691
AKT-pT308	Rabbit		1:1000	CST, 9275
AKT-pT473	Rabbit		1:1000	CST, 4060
GSK3β	Rabbit		1:1000	CST, 12456
GSK3β-pS9	Rabbit		1:1000	CST, 5558
β-actin	Mouse		1:1000	Santa Cruz, sc-47778
Ki67	Rabbit	1:100		Novus, NBP2-19012
